# Accelerometer-derived “weekend warrior” physical activity pattern and all-cause mortality in individuals with kidney stones: a prospective cohort study

**DOI:** 10.3389/fnut.2026.1892920

**Published:** 2026-08-21

**Authors:** Yanhu Meng, Xusheng Yang, Xiaoke Sun, Yatao Jia

**Affiliations:** 1 Department of Urology, Baoji People's Hospital, Baoji, Shaanxi, China; 2 Department of Nephrology, Beijing Rehabilitation Hospital, Affiliated to Capital Medical University, Beijing, China; 3 Department of Urology, Honghui Hospital, Xi'an Jiaotong University, Xi'an, Shaanxi, China

**Keywords:** accelerometer, dose–response, kidney stones, mortality, physical activity, saturation point, weekend warrior

## Abstract

**Background:**

Kidney stone disease is a prevalent urological condition associated with significant metabolic comorbidities and increased mortality risk. While the “weekend warrior” (WW) physical activity pattern has been linked to reduced mortality in the general population, its impact on patients with kidney stones remains unclear. This study aimed to evaluate the association between accelerometer-derived WW physical activity pattern and all-cause mortality in individuals with kidney stones, and to identify the dose–response relationship and optimal activity thresholds.

**Methods:**

We conducted a prospective cohort study using data from the UK Biobank. Participants with a history of kidney stones (*n* = 1,348) were included. Physical activity was measured via wrist-worn accelerometers, categorizing participants into three groups: the regularly active pattern, the WW pattern, and the inactive group. The primary outcome was all-cause mortality. Cox proportional hazards models were used to estimate hazard ratios (HRs) and 95% confidence intervals (CIs), adjusting for demographic, lifestyle, and clinical covariates. Restricted cubic splines (RCS) were employed to explore nonlinear associations.

**Results:**

During a median follow-up of 15.24 years (interquartile range: 5.54–17.36 years), 101 participants died from all causes. Compared with inactive individuals, the WW pattern was associated with a 49% reduction in all-cause mortality (HR 0.51, 95% CI 0.32–0.81) after full adjustment, which was comparable to the benefit observed in regularly active individuals (HR 0.45, 95% CI 0.23–0.90). Dose–response analysis revealed an L-shaped association between total MVPA and all-cause mortality, with a saturation point identified at 183 min/week. Sensitivity analyses demonstrated that the survival benefit of the WW pattern disappeared when the threshold was increased to 230 min/week (HR 0.76, 95% CI 0.47–1.23), a finding consistent with recent high-threshold studies in other cohorts.

**Conclusion:**

The accelerometer-derived WW activity pattern was associated with lower all-cause mortality among individuals with kidney stones, with an association comparable in magnitude to that observed for regular activity. The inverse association between total MVPA and mortality appeared to plateau at approximately 180 min/week; however, this value should not be interpreted as a definitive clinical threshold. These findings support the potential importance of achieving guideline-recommended MVPA, even when concentrated over 1–2 days, and warrant confirmation in prospective and interventional studies.

## 1 Introduction

Kidney stones are a common urological disorder with a rising prevalence worldwide, imposing an increasing public health burden (1). As lifestyle interventions gain prominence in chronic disease management, physical activity—a modifiable risk factor—is receiving growing attention for its potential influence on the long-term prognosis of patients with kidney stones (2, 3). The World Health Organization (WHO) recommends that adults accumulate at least 150 min of moderate-to-vigorous physical activity (MVPA) per week to preserve health (4). In clinical practice, however, many patients struggle to sustain regular exercise habits because of time constraints, which complicates implementation of this guideline.

In recent years, the WW mode—concentrating most weekly MVPA into 1–2 days—has emerged as a flexible exercise strategy (5–8). Objective accelerometer data show that the WW pattern is associated with lower risks of all-cause, cardiovascular, and cancer mortality. Its magnitude of benefit appears comparable to that of regularly distributed activity (9, 10). In populations with chronic conditions, the WW pattern has been shown to reduce mortality risk among patients with cardiovascular disease, metabolic-associated fatty liver disease, and diabetes with chronic kidney disease (11–13). Notably, Ye et al. (14) report that the WW mode is linked to lower risk of chronic kidney disease and acute kidney injury, suggesting potential protective effects on renal outcomes.

Despite increasing evidence linking activity patterns to metabolism and cardiovascular disease, the impact of the WW activity pattern on all-cause mortality among patients with kidney stones remains unexplored. Existing research in this area is constrained by two primary limitations. First, studies have not specifically evaluated mortality risk within the kidney stone population (15). Second, the majority of previous research has relied on self-reported physical activity data rather than objective measures obtained through accelerometers, thereby diminishing the robustness of their conclusions (16, 17). Given that the WW pattern is likely prevalent among individuals with time constraints, and considering the limited attention given to long-term mortality risk in patients with kidney stones, these gaps necessitate urgent investigation.

Previous dose–response studies have shown that higher levels of total physical activity, irrespective of intensity, are associated with a substantially lower risk of premature mortality in middle-aged and older adults, with evidence of a nonlinear dose–response pattern (18). Given that kidney stone disease frequently coexists with metabolic comorbidities and that some groups of stone formers may have an elevated mortality risk (19), determining whether a similar dose–response association exists in this population is clinically relevant. Therefore, this study aimed to objectively characterize the weekend warrior activity pattern among individuals with kidney stones using accelerometer data from the UK Biobank and to examine its association with all-cause mortality. We hypothesized that both the weekend warrior and regularly active patterns would be associated with lower all-cause mortality compared with inactivity and that total MVPA would show a nonlinear inverse association with all-cause mortality.

## 2 Methods

### 2.1 Data source and study population

This research utilized data from the UK Biobank, a substantial prospective, population-based cohort that enrolled over 500,000 participants aged 37 to 73 years throughout the United Kingdom between 2006 and 2010. The UK Biobank amassed comprehensive baseline data, including questionnaire responses, physical measurements, biological samples, and genetic information (20). For the present analysis, the accelerometer subsample collected between 2013 and 2015 was employed (21, 22). The UK Biobank obtained ethical approval from the National Health Service National Research Ethics Service (reference number 11/NW/0382), and this study was conducted under UK Biobank application number 1222281. Participants provided informed consent prior to enrollment. The study was conducted and reported in accordance with Strengthening the Reporting of Observational Studies in Epidemiology (STROBE) guidelines.

**Figure 1:**
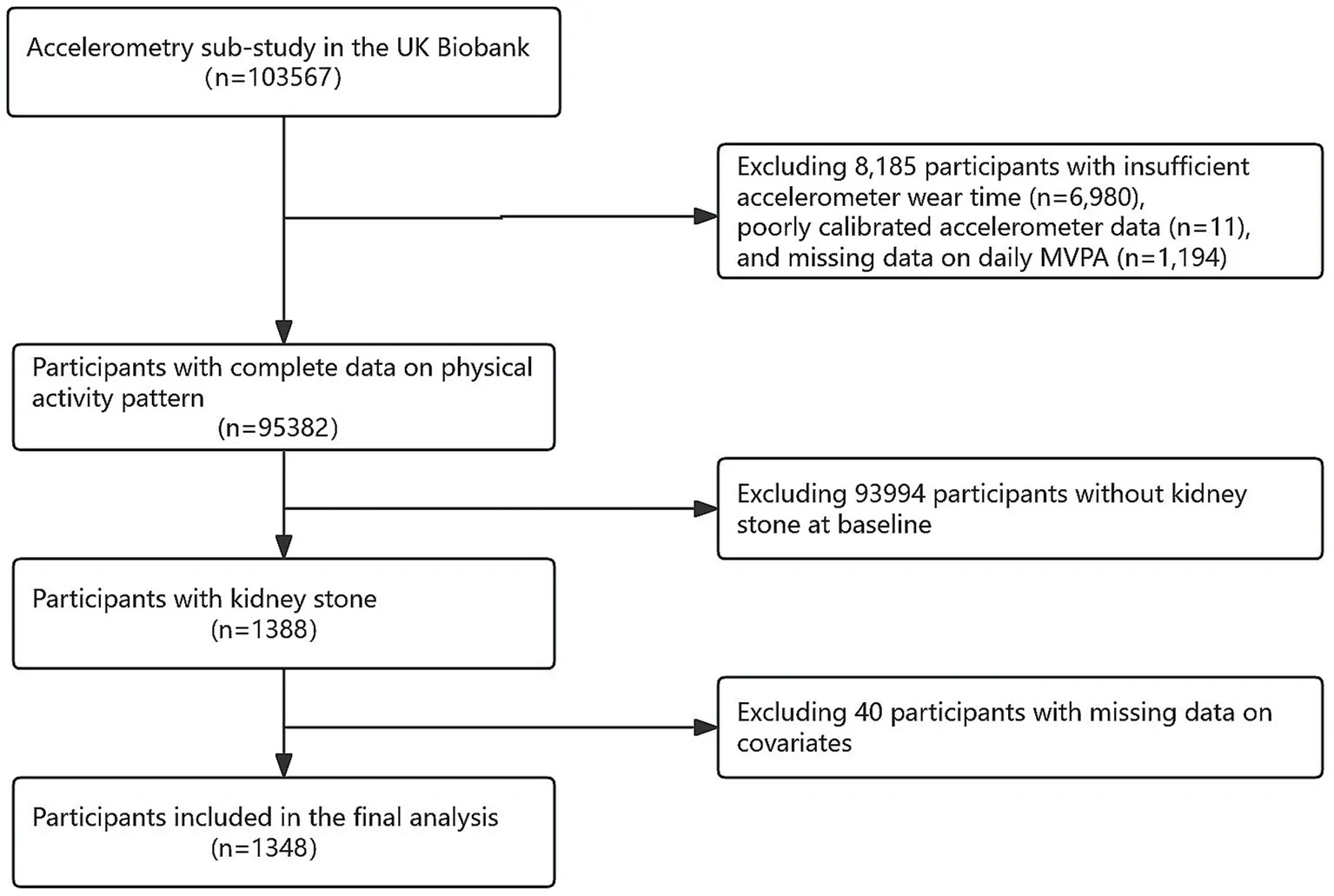
Selection of the study population.

From the UK Biobank accelerometer subsample, we sequentially excluded participants who (1) did not have kidney stones at baseline and (2) had missing data for any covariate. Variables and codes used to define kidney stone disease (as show in Supplementary Table 1). After these exclusions, 1,348 participants with kidney stones remained for the final analysis (Figure 1).

### 2.2 Assessment of physical activity pattern

Physical activity was assessed using an Axivity AX3 triaxial accelerometer (Axivity Ltd., Newcastle upon Tyne, United Kingdom), worn continuously on the dominant wrist for 24 h per day over 7 consecutive days (23, 24). Raw accelerometer signals were calibrated and processed according to the standard UK Biobank protocol.

To ensure data quality, we excluded participants who met any of the following criteria: fewer than 3 days of valid accelerometer data within any 24-h period or any missing data in a continuous 1-h interval (*n* = 6,980); insufficiently calibrated raw accelerometer data (n = 11); or no daily MVPA data (*n* = 1,194).

MVPA was identified using a machine-learning algorithm developed and internally validated by Walmsley et al. using free-living data collected in the CAPTURE-24 study (24, 25). Activity labels derived from wearable-camera images and time-use diaries served as the reference standard. In leave-one-participant-out cross-validation, the algorithm achieved an overall accuracy of 88% and a Cohen’s kappa of 0.80 (24). MVPA duration was derived from UK Biobank field 40,045 (“Moderate-Vigorous—day average”) and expressed as minutes per week. UK Biobank field 40,045 provides the daily proportion of time spent in MVPA for each day from Monday to Sunday. Weekly MVPA duration was calculated as the sum of the seven daily proportions multiplied by 1,440 min/day: weekly MVPA (min/week) = *Σ* (daily MVPA proportion × 1,440). For each participant, daily MVPA values were ranked from highest to lowest, and the sum of MVPA accumulated on the two most active days was divided by total weekly MVPA. Participants were subsequently classified using thresholds of ≥150 min/week and ≥230.4 min/week (26). Participants were classified into three groups: weekend warrior (WW): weekly MVPA ≥150 min and ≥50% of total weekly MVPA accumulated over 1–2 days; Regularly active: weekly MVPA ≥150 min but not meeting the active WW definition; Inactive: weekly MVPA <150 min (4).

### 2.3 Study outcome

The primary outcome was all-cause mortality. Cause-specific mortality was not evaluated in the present study. Mortality data were obtained by linking hospital inpatient records with the national death registration database. Cause of death was determined from codes for underlying cause (field ID: 40001) and/or contributory cause (field ID: 40002) using the International Classification of Diseases, Tenth Revision (ICD-10). Participants were followed from the date of the initial UK Biobank baseline assessment (2006–2010), when kidney stone status and covariates were ascertained, until death or the end of follow-up (November 30, 2024), whichever occurred first. Physical activity was subsequently assessed using 7-day wrist-worn accelerometry during 2013–2015 and was considered an objective indicator of habitual physical activity, supported by evidence of moderate-to-good long-term reproducibility of accelerometer-derived physical activity measures. we also repeated the analyses using the accelerometer wear date as the start of follow-up; the results remained consistent (timeline show as Supplementary Figure 1).

### 2.4 Covariates

Covariates included age (years), sex (male, female), body mass index (kg/m^2^), race/ethnicity (White, non-White), smoking status (never, ever, current), alcohol intake frequency (never, ≤1–2 times/week, ≥3 times/week), Townsend deprivation index, educational level (college or university degree, other professional qualifications), water intake, serum creatinine, urinary potassium, urinary sodium, cholesterol, triglycerides, and total protein. Detailed definitions and categorizations are provided in Supplementary Table 2. Covariate data were taken from the baseline UK Biobank assessment or from the first 24-h dietary recall interview.

### 2.5 Statistical analysis

To evaluate the normality of variables, we employed histogram inspection, Q–Q plots, and the Kolmogorov–Smirnov test. Continuous variables following a normal distribution are presented as mean ± standard deviation (SD), whereas non-normally distributed continuous variables are reported as median and interquartile range (IQR). Categorical variables are described using frequencies and percentages (%).

Based on the results of normality assessment, continuous variables were compared between groups using either the independent-samples T test or the Mann–Whitney U test. Categorical data were analyzed with the chi-square test or Fisher’s exact test, as appropriate.

HRs and 95% CIs for all-cause mortality in relation to the accelerometer-derived WW physical activity pattern were estimated using Cox proportional hazards models. Both unadjusted and multivariable-adjusted models were fitted to evaluate the association.

All-cause mortality was evaluated with Kaplan–Meier survival curves stratified by MVPA and compared using the log-rank test. MVPA was modeled as a categorical variable using the guideline-based threshold and the sample-median threshold. Confounders were selected based on clinical judgment and a review of the relevant literature.

We constructed a series of models to assess potential confounding. Model 1 was unadjusted. Model 2 adjusted for age, sex, race, and ethnicity. Model 3 additionally adjusted for body mass index (BMI), Townsend deprivation index, smoking status, frequency of alcohol consumption, and education level. Sensitivity analyses were performed by extra adjustment of the fully confounder-adjusted model for water consumption, serum creatinine, urinary potassium, urinary sodium, total cholesterol, triglycerides, and serum total protein. This structured approach ensures a comprehensive analysis of the data while controlling for various confounding factors to enhance the reliability of the findings.

To examine the nonlinear relationship between MVPA and all-cause mortality among participants with kidney stones, we employed restricted cubic splines (RCS). Based on the results of the smoothing curve, we subsequently applied a two-piecewise linear regression model to identify potential threshold effects, while controlling for possible confounding variables.

Subgroup analyses were conducted according to predefined subgroup variables, and interactions among these subgroups were evaluated using the likelihood ratio test. Missing data, comprising less than 5% of the entire dataset, were addressed through deletion for the primary analysis. Furthermore, to enhance statistical power and reduce potential bias due to missing data, we implemented multiple imputation as a sensitivity analysis. This approach used five imputations with the multivariate imputation by chained equations method, as previously described (27).

To evaluate the robustness of our findings, we performed a series of sensitivity analyses to investigate the impact of employing alternative association inference models on our conclusions. We present and compare the effect sizes and *p*-values generated by each model.

Statistical analyses were performed using R statistical software (version 4.2.2). All statistical tests were two-sided, with *p* < 0.05 denoting statistical significance.

## 3 Results

### 3.1 Baseline characteristics of participants

The final analytic cohort included 1,348 individuals with kidney stones, with a mean age of 57.9 years (SD, 7.3); 31.4% were female. Of these participants, 565 (41.9%) were classified as WW, 241 (17.9%) as regularly active, and 542 (40.2%) as inactive.

Baseline characteristics according to physical activity pattern are presented in Table 1. Compared with inactive participants, those in the WW and regularly active groups tended to be younger, more often male, have lower body weight and higher educational attainment, and reported drinking alcohol over three times weekly. Weekly MVPA was concentrated within the two most active days in the WW group but was distributed more evenly throughout the week in the regularly active group.

**Table 1:** Baseline characteristics of participants with kidney stones in the final analytic cohort.

**Characteristics**	**Total population**	**Physical activity patterns**			***P value***
		Inactive	Regularly active	Weekend warrior	
*N* (%)	1348 (100)	542 (40.2)	241 (17.9)	565 (41.9)	
Age, mean (SD), y	57.9 ± 7.3	58.4 ± 7.4	57.0 ± 7.5	57.7 ± 7.2	0.034
Sex, *n* (%)					< 0.001
Female	423 (31.4)	214 (39.5)	69 (28.6)	140 (24.8)	
Male	925 (68.6)	328 (60.5)	172 (71.4)	425 (75.2)	
BMI, mean (SD), kg/m2	28.0 ± 4.8	29.3 ± 5.4	27.1 ± 4.1	27.1 ± 4.1	< 0.001
Race and ethnicity, *n* (%)					0.304
White	1318 (97.8)	526 (97)	236 (97.9)	556 (98.4)	
Non-white	30 (2.2)	16 (3)	5 (2.1)	9 (1.6)	
Smoking status, *n* (%)					0.077
Never smoker	717 (53.2)	273 (50.4)	129 (53.5)	315 (55.8)	
Former smoker	513 (38.1)	208 (38.4)	96 (39.8)	209 (37)	
Current smoker	118 (8.8)	61 (11.3)	16 (6.6)	41 (7.3)	
Frequency of alcohol drinking, *n* (%)					0.021
Never	93 (6.9)	49 (9)	15 (6.2)	29 (5.1)	
≤1-2 times/week	652 (48.4)	273 (50.4)	106 (44)	273 (48.3)	
≥3 times/week	603 (44.7)	220 (40.6)	120 (49.8)	263 (46.5)	
Education, *n* (%)					< 0.001
College or university degree	564 (41.8)	177 (32.7)	115 (47.7)	272 (48.1)	
Others	784 (58.2)	365 (67.3)	126 (52.3)	293 (51.9)	
Townsend deprivation index, median (IQR)	-2.3 (-3.7, 0.0)	-2.3 (-3.7, 0.2)	-1.8 (-3.6, 0.9)	-2.4 (-3.8, -0.5)	0.022
Water intake, median (IQR), Glass/Beaker	2.0 (1.0, 4.0)	2.0 (1.0, 4.0)	3.0 (1.0, 4.0)	2.0 (1.0, 4.0)	0.042
Blood Creatinine, mean (SD), umol/L	76.5 ± 16.5	76.1 ± 19.1	76.8 ± 15.3	76.8 ± 14.2	0.801
Potassium in urine, mean (SD), millimole/L	65.0 ± 32.0	63.6 ± 30.8	67.7 ± 34.2	65.1 ± 32.1	0.266
Sodium in urine, mean (SD), millimole/L	81.7 ± 44.6	83.6 ± 45.5	81.5 ± 47.5	79.8 ± 42.5	0.371
Cholesterol, mean (SD), mmol/L	5.6 ± 1.2	5.5 ± 1.2	5.7 ± 1.2	5.5 ± 1.1	0.378
Triglycerides, mean (SD), mmol/L	1.9 ± 1.1	2.1 ± 1.2	1.8 ± 1.0	1.9 ± 1.1	< 0.001
Total protein, mean (SD), g/L	72.1 ± 4.0	71.9 ± 3.8	72.6 ± 4.2	72.2 ± 3.9	0.07
Total MVPA, median (IQR), min	198.4 (85.8, 383.3)	70.5 (28.8, 100.8)	432.0 (329.6, 604.8)	288.0 (213.1, 436.2)	< 0.001

*BMI*, body mass index; *SD*, standard deviation. *IQR* interquartile range.

Data are presented as number (proportion) or mean (SD).

Weekend warrior pattern was defined as MVPA ≥ 150 min/week (guideline-recommended) with ≥ 50% concentrated on 1–2 days per week; regularly active pattern was defined as MVPA ≥ 150 min/week with ≥ 50% distributed on > 2 days per week; inactive group was defined as MVPA < 150 min/week.

### 3.2 Associations of physical activity patterns with all-cause mortality

During a median follow-up of 15.24 years from the initial recruitment visit, 101 participants died. In the sensitivity analysis using the accelerometer wear date as the start of follow-up, the median follow-up was 9.74 years.

**Figure 2:**
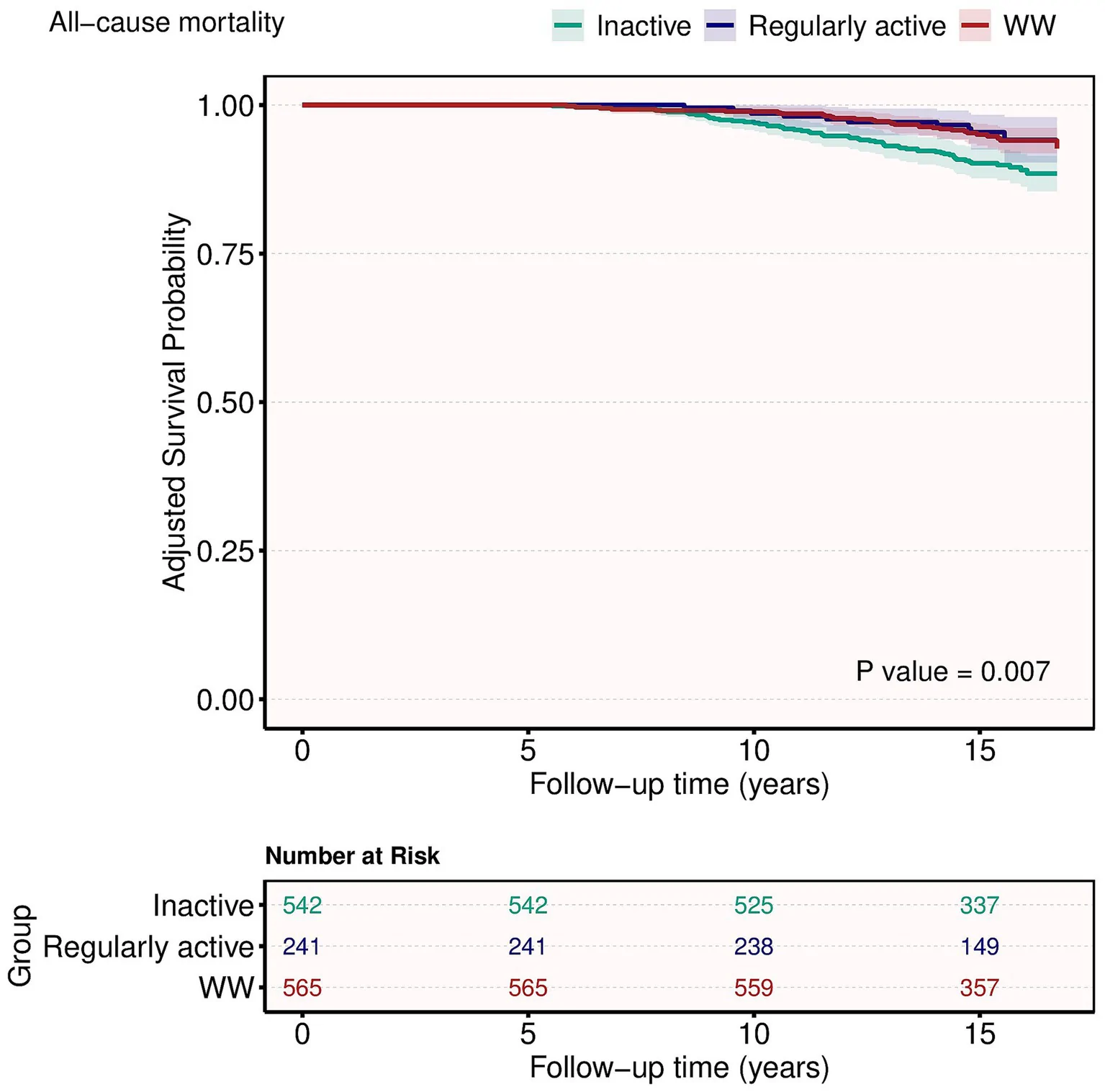
Adjusted survival curves for all-cause mortality according to physical activity pattern. WW pattern was defined as MVPA ≥ 150 min/week (guideline-recommended) with ≥ 50% concentrated on 1–2 days per week; regularly active pattern was defined as MVPA ≥ 150 min/week with ≥ 50% distributed on > 2 days per week; inactive group was defined as MVPA < 150 min/week. *Abbreviation*: *MVPA*: moderate-to-vigorous intensity physical activity; Adjusted for age, sex, ethnicity, Townsend deprivation index, education, smoking status, alcohol drinking, BMI.

Survival probabilities differed across the three physical activity patterns (log-rank *p* = 0.004; Figure 2). In the fully adjusted model, compared with the inactive group, both the WW pattern (HR, 0.51; 95% CI, 0.32–0.81) and the regularly active pattern (HR, 0.45; 95% CI, 0.23–0.90) were associated with lower all-cause mortality (Table 2). The point estimates were similar in magnitude. No significant difference was observed between the WW and regularly active groups (HR, 1.14; 95% CI, 0.55–2.33; Supplementary Table 3).

**Table 2:** Associations between physical activity patterns and all-cause mortality in individuals with kidney stone.

Outcome	Total	*N* event%	Model 1		Model 2		Model 3	
			HR (95%CI)	*P value*	HR (95%CI)	*P value*	HR (95%CI)	*P value*
Inactive	542	61 (11.3)	1.00 (Reference)	…	1.00 (Reference)	…	1.00 (Reference)	…
Regularly active	241	10 (4.1)	0.36 (0.19 ~ 0.71)	0.003	0.37 (0.19 ~ 0.73)	0.004	0.45 (0.23 ~ 0.9)	0.023
Weekend warrior	565	30 (5.3)	0.46 (0.3 ~ 0.71)	0.001	0.44 (0.28 ~ 0.69)	<0.001	0.51 (0.32 ~ 0.81)	0.004
Trend test	1,348	101 (7.5)	0.66 (0.52 ~ 0.82)	<0.001	0.65 (0.52 ~ 0.81)	<0.001	0.71 (0.56 ~ 0.89)	0.004

Weekend warrior pattern was defined as MVPA ≥ 150 min/week (guideline-recommended) with ≥ 50% concentrated on 1–2 days per week; regularly active pattern was defined as MVPA ≥ 150 min/week with ≥ 50% distributed on > 2 days per week; inactive group was defined as MVPA < 150 min/week. Model 1: unadjusted. Model 2: adjusted for age, sex, race and ethnicity. Model 3: adjusted for model 2 + BMI, Townsend deprivation index, Smoking status, Frequency of alcohol drinking, education.

### 3.3 Alternative MVPA threshold and dose–response analysis

When activity was defined using the sample-median threshold of ≥230 min/week, the association between the WW pattern and all-cause mortality was attenuated and no longer statistically significant (Supplementary Table 4).

**Figure 3:**
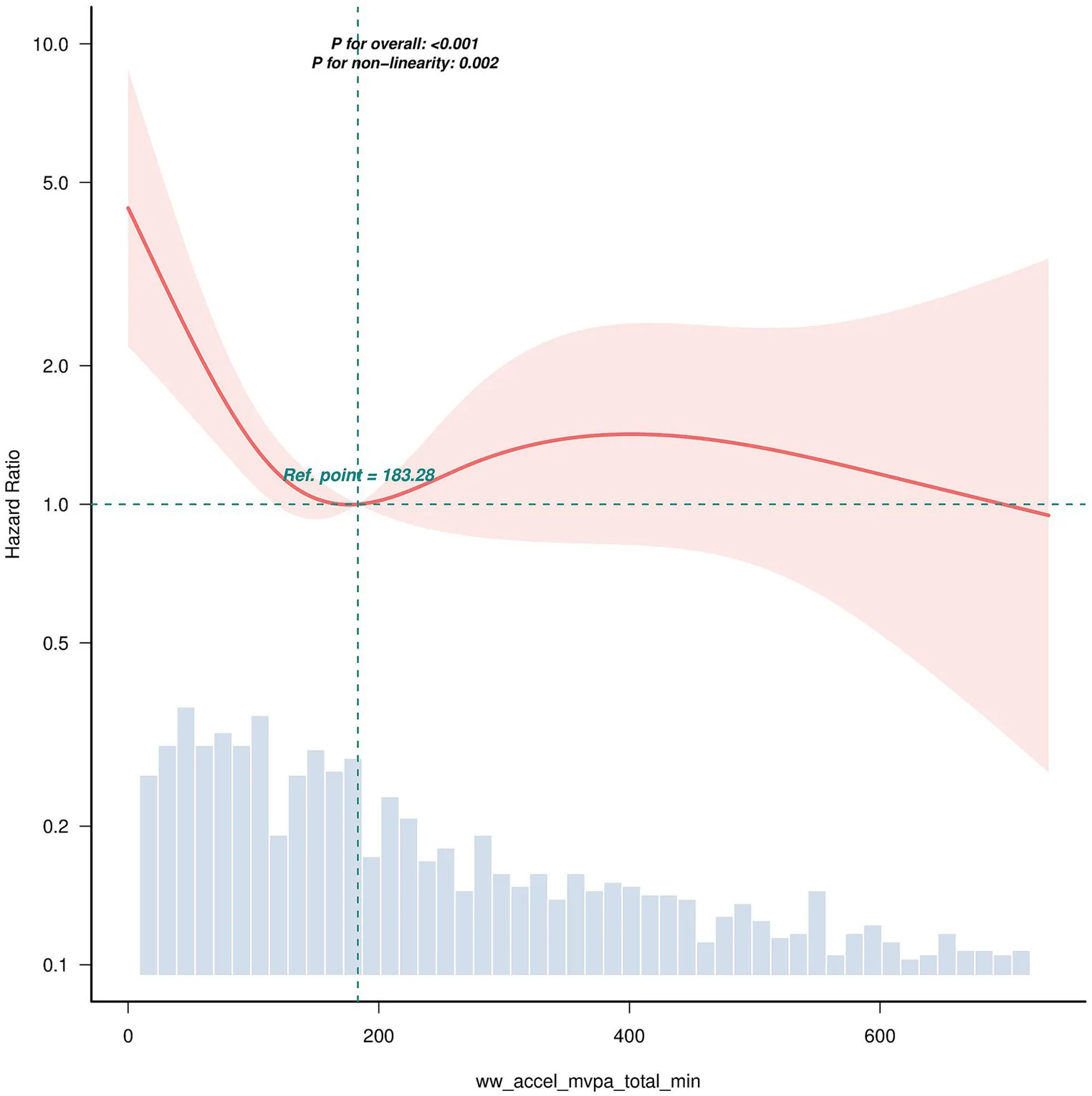
Restricted cubic spline analysis of total MVPA and all-cause mortality. The red solid line illustrates the smoothed curve fit between the variables, with the pink dashed lines and the area between them indicate 95% confidence intervals around the fitted values. Adjusted for age, sex, ethnicity, Townsend deprivation index, education, smoking status, alcohol drinking, BMI.

Restricted cubic spline analysis showed a nonlinear L-shaped association between total MVPA and all-cause mortality (*P* for nonlinearity < 0.05; Figure 3). Mortality risk decreased markedly with increasing MVPA up to approximately 183 min/week, after which the association appeared to plateau (Table 3). However, this value should not be interpreted as a definitive biological or clinical threshold and requires confirmation in prospective and interventional studies.

**Table 3:** Association between total MVPA and all-cause mortality in individuals with kidney stone using two-piecewise regression models.

Total MVPA	Crude model		Adjusted model*	
	HR (95%CI)	*P-value*	HR (95%CI)	*P-value*
<183 min	0.991 (0.986,0.996)	< 0.001	0.992 (0.987,0.997)	0.003
≥183 min	1 (0.998,1.002)	0.974	1 (0.998,1.001)	0.795

HR, hazard ratio; 95% CI, 95% confidence interval; MVPA, moderate-to-vigorous intensity physical activity. * Adjusted for age, sex, ethnicity, Townsend deprivation index, education, smoking status, alcohol drinking, BMI.

### 3.4 Subgroup analyses and sensitivity analyses

**Figure 4:**
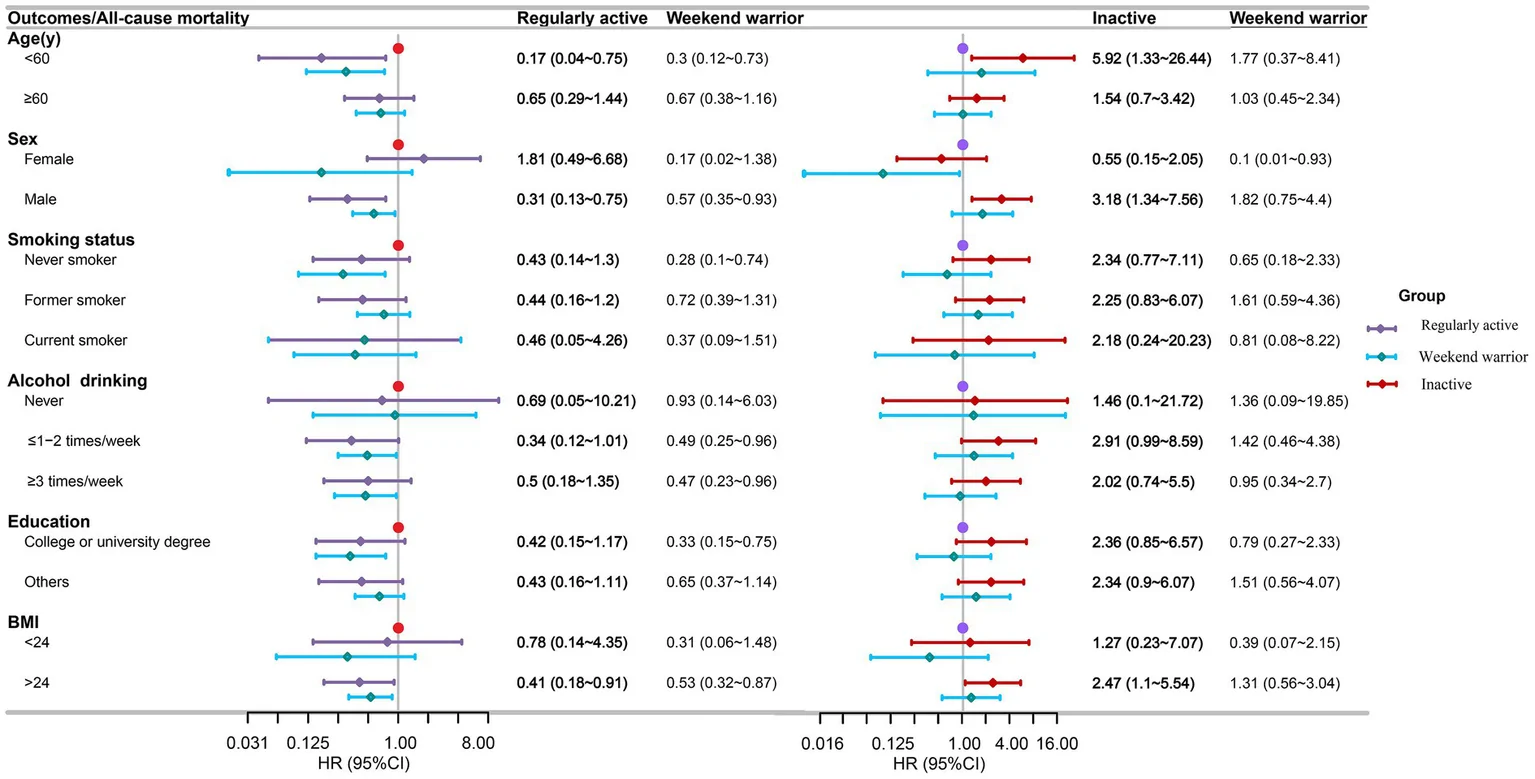
Associations between different physical active patterns and all-cause mortality in patients with kidney stones stratified by age, sex, smoking status, alcohol consumption frequency, education and BMI. WW pattern was defined as MVPA ≥ 150 min/week (guideline-recommended) with ≥ 50% concentrated on 1–2 days per week; regularly active pattern was defined as MVPA ≥ 150 min/week with ≥ 50% distributed on > 2 days per week; inactive group was defined as MVPA < 150 min/week. The left panel uses the inactive group as the reference; the right panel uses the regularly active group as the reference. MVPA: moderate-to-vigorous intensity physical activity; BMI, body mass index; HR: hazard ratio; CI: confidence interval.

Subgroup analyses according to age, sex, smoking status, alcohol consumption, education, and BMI are presented in Figure 4 and Supplementary Table 5. Among participants aged <60 years, male individuals, and those with a BMI > 24, both the regularly active pattern (HR, 0.17; 95% CI, 0.04–0.75) and the WW pattern (HR, 0.30; 95% CI, 0.12–0.73) were associated with lower all-cause mortality compared with inactivity.

The associations varied by sex. Among females, neither the regularly active pattern (HR, 1.81; 95% CI, 0.49–6.68) nor the WW pattern (HR, 0.17; 95% CI, 0.02–1.38) was significantly associated with all-cause mortality. These estimates had wide confidence intervals and should therefore be interpreted cautiously. Evidence of interaction was observed for sex (*P* for interaction = 0.031, Supplementary Table 5), whereas no significant interactions were observed for the other subgroup variables.

After additional adjustment for water intake, serum creatinine, urinary potassium, urinary sodium, cholesterol, triglycerides, and total protein, the association for the WW group remained statistically significant, whereas that for the regularly active group was attenuated (Supplementary Table 6). Results from multiple imputation analyses were consistent with the primary findings (Supplementary Table 7).

In analyses stratified by baseline kidney stone status, both the regularly active pattern (HR, 0.46; 95% CI, 0.23–0.91) and the WW pattern (HR, 0.51; 95% CI, 0.32–0.82) were associated with lower all-cause mortality among individuals with kidney stones. However, the interaction between physical activity pattern and baseline kidney stone status was not statistically significant (*P* for interaction = 0.314), indicating no clear evidence that the associations differed according to kidney stone status (Supplementary Table 8). The results also remained materially unchanged when follow-up began at the accelerometer wear date (Supplementary Table 9 and Supplementary Figure 2) and after excluding deaths occurring within 1 and 2 years after accelerometer assessment (Supplementary Tables 10, 11).

## 4 Discussion

### 4.1 Principal findings

Using data from the UK Biobank, this study examined the association between accelerometer-derived physical activity patterns and all-cause mortality among individuals with a history of kidney stones. To our knowledge, this is the first large-scale prospective study to evaluate the WW pattern in this population using device-measured physical activity.

At the guideline-based threshold of 150 min/week, both the WW and regularly active patterns were associated with lower all-cause mortality compared with inactivity. After multivariable adjustment, the WW pattern was associated with an HR of 0.51 (95% CI, 0.32–0.81), while the corresponding HR for the regularly active pattern was 0.45 (95% CI, 0.23–0.90). Thus, the associations observed for the two active patterns were similar in magnitude. However, our analysis did not formally establish equivalence between the two patterns.

Restricted cubic spline analysis suggested a nonlinear L-shaped association between total MVPA and all-cause mortality. Mortality risk declined markedly as MVPA increased up to approximately 183 min/week, after which the association appeared to plateau. This value should be interpreted as an exploratory point on the dose–response curve rather than a definitive biological or clinical threshold. When the activity threshold was increased to the sample median of 230.4 min/week, the association for the WW pattern was attenuated and no longer statistically significant (HR, 0.76; 95% CI, 0.47–1.23). However, this finding should not be interpreted as evidence of harm or the absence of an association at higher activity levels. Changing the threshold altered the composition and size of the activity groups and reduced the precision of the estimate, and statistical non significance alone does not demonstrate the absence of an association (28).

### 4.2 Comparison with previous studies

Our findings are consistent with large cohort studies based on accelerometer-derived physical activity. Ren et al. and Liao et al. reported that both WW and regularly active patterns were associated with lower all-cause mortality in the general population (9, 29). Similar associations have also been reported among individuals with cardiovascular disease, hypertension, cancer, and type 2 diabetes (11, 30–32). Collectively, these studies suggest that achieving a sufficient total volume of MVPA may be more relevant to mortality risk than distributing activity evenly throughout the week. Nevertheless, differences in activity definitions, thresholds, study populations, and follow-up periods should be considered when comparing estimates across studies.

The present study extends previous research on the WW pattern, which has primarily focused on populations with cardiovascular disease, hypertension, diabetes, cancer, or depression (33–37). Kidney stone disease is a recurrent condition that frequently coexists with obesity, diabetes, hypertension, dyslipidemia, and other features of metabolic syndrome (38, 39). The demographic profile in this study also differs notably from prior reports. Kidney stone patients are generally younger, predominantly male (70–80%), and largely employed, whereas earlier studies reported higher proportions of middle-aged and elderly individuals and of women (40–42). Previous investigations of physical activity and kidney stones have mainly examined stone occurrence or recurrence. For example, Liu et al. reported an inverse association between total physical activity and kidney stone disease, while Sorensen et al. found that physical activity was associated with a lower risk of incident stones among postmenopausal women (43, 44). Finally, methodological differences between studies must be acknowledged. Previous investigations of WW relied largely on questionnaires, which introduced substantial recall bias and were susceptible to the “healthy worker effect” (32, 45, 46). In contrast, this study used wrist-worn accelerometers for objective measurement, markedly improving data accuracy. By examining all-cause mortality among individuals with an established history of kidney stones, our study broadens the focus from stone prevention to longer-term health outcomes.

The findings at different activity thresholds may also help contextualize heterogeneity in the existing literature. Ren et al. reported that associations for the WW pattern became less consistent when activity was defined using a higher threshold of approximately 230 min/week (29). Wang et al. similarly observed attenuated associations for some mortality outcomes when a substantially higher MVPA threshold was applied among individuals with cardiovascular disease (11). These findings do not establish a universal saturation point but indicate that estimates for activity patterns can depend on the threshold used to define sufficient activity. Additional studies are needed to characterize the dose–response association and assess whether it differs according to population characteristics and clinical status.

### 4.3 Interpretation and potential mechanisms

The similar associations observed for the weekend warrior and regularly active patterns suggest that total weekly MVPA volume may be more important than its distribution across the week. When a comparable amount of MVPA is accumulated, longer or more concentrated activity sessions within 1–2 days may still provide sufficient physiological stimuli to produce sustained improvements in cardiorespiratory fitness, insulin sensitivity, glucose regulation, blood pressure, systemic inflammation, and oxidative stress (47–49). Because some physiological responses to exercise persist beyond an individual activity session, their cumulative effects may not require MVPA to be performed every day. This may partly explain why concentrated and regularly distributed activity patterns were associated with similarly lower mortality risk. However, our observational data cannot establish that the two activity patterns produce equivalent physiological effects.

These mechanisms may be particularly relevant to individuals with kidney stones because insulin resistance and metabolic abnormalities commonly coexist with stone disease (39, 44, 47). Exercise-related improvements in adiposity and blood pressure may also influence the urinary biochemical environment, although evidence regarding direct effects on urinary calcium, sodium, and uric acid excretion remains limited (44, 48). Because these potential mediators were not directly assessed in our study, the proposed mechanisms remain speculative and require confirmation in mechanistic studies.

The restricted cubic spline analysis suggested that the inverse association between total MVPA and all-cause mortality became less pronounced beyond approximately 183 min/week. This apparent plateau may reflect diminishing marginal reductions in mortality risk after an adequate weekly activity volume has been accumulated. It may also be influenced by greater statistical uncertainty at higher MVPA levels and the data-dependent nature of spline modelling. Therefore, 183 min/week should be interpreted as an exploratory feature of the observed dose–response curve rather than a definitive biological threshold or an optimal exercise prescription.

From a practical perspective, accumulating guideline-recommended MVPA within 1–2 days may represent a feasible alternative for individuals who have difficulty exercising regularly throughout the week. Nevertheless, the findings should not be interpreted as evidence that concentrated activity causes lower mortality or as support for prescribing approximately 183 min/week as an optimal target. Prospective studies with repeated activity assessments and randomized intervention trials are needed before specific clinical recommendations can be made.

### 4.4 Strengths and limitations

This study has several strengths. Physical activity was objectively assessed using wrist-worn accelerometers, reducing the recall and social desirability biases associated with questionnaire-based measurements. The analyses also considered both a guideline-recommended threshold and a data-derived higher threshold and used restricted cubic splines to characterize the continuous dose–response association. Furthermore, the extensive covariate data available in the UK Biobank allowed multivariable adjustment and several sensitivity analyses.

Several limitations should also be considered. First, selection bias and limited generalizability remain concerns. Only approximately 5.5% of individuals invited to participate in the UK Biobank completed the baseline assessment, and participants were generally healthier and less socioeconomically deprived than the source population. The cohort was also predominantly White. Therefore, the findings may not be directly generalizable to more diverse populations or individuals with lower socioeconomic status (50).

Second, physical activity was measured over only 7 days and was assumed to represent habitual activity. Previous repeat-measurement evidence has indicated moderate-to-good stability of accelerometer-derived activity over approximately 3.7 years (51), but changes in activity behavior during follow-up could still result in exposure misclassification. In addition, covariates were collected during the initial UK Biobank assessment, whereas accelerometer measurements were obtained several years later. The primary analysis calculated follow-up from the initial recruitment visit, which may introduce exposure misclassification and potential immortal time bias during the pre-accelerometer period. Nevertheless, the results remained materially unchanged in the sensitivity analysis in which follow-up was recalculated from the accelerometer wear date.

Third, residual confounding and reverse causation cannot be completely excluded. Although we adjusted for a wide range of covariates—including water intake, serum creatinine, urinary potassium, urinary sodium, cholesterol, triglycerides, and total protein—and obtained consistent results after excluding deaths within 1 and 2 years after accelerometer assessment, the inactive group may still have had poorer underlying health. Information on certain comorbidities, chronic kidney disease (CKD)/ estimated glomerular filtration rate (eGFR), sedentary time, stone severity, treatment history, detailed dietary factors, and genetic susceptibility was not fully captured. Unmeasured or incompletely measured confounding may therefore have influenced the observed associations. Finally, the relatively small number of deaths limited the precision of estimates, particularly in analyses using higher activity thresholds and in subgroup analyses.

## 5 Conclusion

In this prospective cohort study, an accelerometer-derived WW physical activity pattern was associated with lower all-cause mortality among individuals with kidney stones (HR, 0.51; 95% CI, 0.32–0.81), with a point estimate similar in magnitude to that observed for the regularly active pattern. Restricted cubic spline analysis suggested a nonlinear L-shaped association between total MVPA and all-cause mortality, with the risk reduction appearing to plateau at approximately 183 min/week. However, this value should not be interpreted as a definitive clinical threshold, and the nonsignificant association observed at the 230 min/week cutoff should be interpreted cautiously. Overall, these findings suggest that accumulating guideline-recommended MVPA, even when concentrated within 1–2 days, may be associated with lower long-term mortality risk in individuals with kidney stones. Further prospective and interventional studies are needed to confirm these findings and determine their clinical relevance.

## Data Availability

The dataset supporting the conclusions of this article is available via the UK BioBank. Access to the UK Biobank resource can be obtained via an approved application (https://www.ukbiobank.ac.uk/use-our-data/apply-for-access). Further enquiries can be directed to the corresponding author.
