# Supplementary material for: Accelerometer-derived “weekend warrior” physical activity pattern and all-cause mortality in individuals with kidney stones: a prospective cohort study
Source: Front Nutr. 2026 Aug 21;13:1892920. doi: 10.3389/fnut.2026.1892920 (PMC13541727; doi:10.3389/fnut.2026.1892920)
Supplement: Supplementary file 1 [file Table_1.pdf]

Accelerometer-derived "weekend warrior" physical activity pattern and all-cause mortality in individuals with kidney stones: a prospective cohort study

Yanhu Meng [1]✝, Xusheng Yang[2]✝, Xiaoke Sun[3], Yatao Jia[3]*

Supplementary Table 1: Variables and codes used to define kidney stone disease in UK Biobank study

Outcome	ICD-10	Self-reported field (Coding 6)
Kidney stone	N20, N200, N202, N209	1197

Abbreviation: ICD10: the 10th vision of International Classification of Diseases

Supplementary Table 2: Definitions and codes of covariates

Variable	File ID	File names	Definition and categories in this study
Age	21022	Age at recruitment	This is a derived variable based on date of birth and date of attending an initial assessment center
Sex	31	Sex	Women and men.
Ethnicity	21000	Ethnic background	Ethnicity was self-reported and classified into white and non-white.
Townsend deprivation index	22189	Townsend deprivation index at recruitment	A comprehensive indicator of socioeconomic status based on unemployment, non-car ownership, non-cottage ownership, and household overcrowding. A higher value indicates greater deprived.

Education	6138	Education qualifications	Self-reported education was classified into college or university degree and others professional qualifications.
Smoking status	20116	Smoking status	Smoking status was assessed using self-reported questionnaire and categorized into never, ever, and current.
Frequency of alcohol drinking	1558	Alcohol intake frequency	Frequency of alcohol drinking was assessed using self-reported questionnaire and categorized into never, ≤1-2 times a week, and ≥3 times a week.
Body mass index (BMI)	23104	Body mass index	BMI (kg/m^2) was calculated by dividing the weight in kilograms (kg) by the square of height in meters (m).
Water intake	1528	Water intake	How many glasses of water do you drink each DAY?（Glass/Beaker）
Blood Creatinine	30700	Creatinine	Measured by enzymatic analysis on a Beckman Coulter AU5800（umol/L）
Potassium in urine	30520	Potassium in urine	Measured by ISE (ion selective electrode) analysis on a Beckman Coulter AU5400（millimole/L）
Sodium in urine	30530	Sodium in urine	Measured by ISE (ion selective electrode) analysis using Beckman Coulter AU5400（millimole/L）
Cholesterol	30690	Cholesterol	Measured by CHO-POD analysis on a Beckman Coulter AU5800（ mmol/L）
Triglycerides	30870	Triglycerides	Measured by GPO-POD analysis on a Beckman Coulter AU5800（mmol/L）

Total protein	30860	Total protein	Measured by biuret analysis on a Beckman Coulter AU5800（g/L）

Supplementary Table 3: Association between physical activity pattern and all-cause mortality in individuals with kidney stones

Outcome	Physical activity pattern	Model1	Model2	Model3
		HR(95％CI)	HR(95％CI)	HR(95％CI)
All-cause mortality	Regularly active	1.00 (Reference)	1.00 (Reference)	1.00 (Reference)
	Inactive	2.75 (1.41~5.38)	2.68 (1.37~5.25)	2.22 (1.12~4.4)
	Weekend warrior	1.27 (0.62~2.59)	1.19 (0.58~2.43)	1.14 (0.55~2.33)

HR, hazard ratio; *CI*: confidence interval

Weekend warrior pattern was defined as MVPA ≥ 150 min/week (guideline-recommended) with ≥ 50% concentrated on 1–2 days per week; regularly active pattern was defined as MVPA ≥ 150 min/week with ≥ 50% distributed on > 2 days per week; inactive group was defined as MVPA < 150 min/week

Model 1: unadjusted.

Model 2: adjusted for age, sex, race and ethnicity.

Model 3: adjusted for model 2+ BMI, Townsend deprivation index, Smoking status, Frequency of alcohol drinking, education

Supplementary Table 4: Association between physical activity pattern and all-cause mortality in individuals with kidney stone disease (weekend warrior pattern was defined as MVPA ≥230 min/week with ≥50% concentrated on 1-2 days per week)

Outcome	Total	N event%	Model1		Model2		Model3	
			HR(95％CI)	P value	HR(95％CI)	P value	HR(95％CI)	P value

Inactive	752	68 (9)	1.00 (Reference)	…	1.00 (Reference)	…	1.00 (Reference)	…
Regularly active	219	9 (4.1)	0.45 (0.23~0.91)	0.025	0.46 (0.23~0.92)	0.028	0.56 (0.28~1.14)	0.109
Weekend warrior	377	24 (6.4)	0.7 (0.44~1.11)	0.126	0.65 (0.41~1.04)	0.073	0.76 (0.47~1.23)	0.259
Trend test	1348	101 (7.5)	0.8 (0.63~1.01)	0.064	0.77 (0.61~0.99)	0.039	0.85 (0.66~1.09)	0.191

HR, hazard ratio; *CI*: confidence interval; *MVPA*, moderate to vigorous physical activity;

Weekend warrior pattern was defined as MVPA ≥ 150 min/week (guideline-recommended) with ≥ 50% concentrated on 1–2 days per week; regularly active pattern was defined as MVPA ≥ 150 min/week with ≥ 50% distributed on > 2 days per week; inactive group was defined as MVPA < 150 min/week

Model 1: unadjusted.

Model 2: adjusted for age, sex, race and ethnicity.

Model 3: adjusted for model 2+ BMI, Townsend deprivation index, Smoking status, Frequency of alcohol drinking, education

Supplementary Table 5: Association between physical activity pattern and all-cause mortality in individual with kidney stones, stratified by age, sex, race and ethnicity, BMI, Townsend deprivation index, Smoking status, Frequency of alcohol drinking, education

Subgroup	Variable	Total	N event%	Follow up Time (per 1000 person-year)	HR(95%CI)	*P value*	*P for interaction*
Age(y)							0.241
< 60							
	Inactive	253	18 (7.1)	4.6	1(Ref)		
	Regularly active	130	2 (1.5)	1	0.17 (0.04~0.73)	0.017	
	Weekend warrior	300	8 (2.7)	1.7	0.31 (0.13~0.75)	0.009	

	Trend test	683	28 (4.1)	2.7	0.52 (0.33~0.84)	0.007	
≥60							
	Inactive	289	43 (14.9)	9.9	1(Ref)		
	Regularly active	111	8 (7.2)	4.7	0.63 (0.29~1.37)	0.241	
	Weekend warrior	265	22 (8.3)	5.5	0.62 (0.36~1.06)	0.082	
	Trend test	665	73 (11)	7.3	0.78 (0.6~1.03)	0.079	
Sex							0.031
Female							
	Inactive	214	12 (5.6)	3.7	1(Ref)		
	Regularly active	69	4 (5.8)	3.8	1.7 (0.5~5.79)	0.396	
	Weekend warrior	140	1 (0.7)	0.5	0.15 (0.02~1.17)	0.071	
	Trend test	423	17 (4)	2.6	0.54 (0.27~1.09)	0.087	
Male							
	Inactive	328	49 (14.9)	10.0	1(Ref)		
	Regularly active	172	6 (3.5)	2.3	0.31 (0.13~0.73)	0.008	
	Weekend warrior	425	29 (6.8)	4.5	0.56 (0.35~0.91)	0.02	
	Trend test	925	84 (9.1)	6.0	0.74 (0.57~0.95)	0.019	
Smoking status							0.355
Never smoker							
	Inactive	273	20 (7.3)	4.8	1(Ref)		
	Regularly active	129	4 (3.1)	2.0	0.45 (0.15~1.37)	0.161	
	Weekend warrior	315	6 (1.9)	1.2	0.31 (0.12~0.79)	0.015	
	Trend test	717	30 (4.2)	2.7	0.54 (0.34~0.87)	0.012	
Former smoker							
	Inactive	208	28 (13.5)	9.0	1(Ref)		
	Regularly active	96	5 (5.2)	3.4	0.44 (0.17~1.18)	0.103	

		Weekend warrior	209	21 (10)	6.6	0.68 (0.37~1.23)	0.2	
		Trend test	513	54 (10.5)	7.0	0.82 (0.6~1.12)	0.212	
	Current smoker							
		Inactive	61	13 (21.3)	14.7	1(Ref)		
		Regularly active	16	1 (6.2)	4.2	0.38 (0.05~3.18)	0.375	
		Weekend warrior	41	3 (7.3)	4.8	0.38 (0.1~1.48)	0.164	
		Trend test	118	17 (14.4)	9.7	0.6 (0.3~1.2)	0.148	
Alcohol drinking								0.974
	Never							
		Inactive	49	6 (12.2)	8.1	1(Ref)		
		Regularly active	15	1 (6.7)	4.4	0.6 (0.05~7.92)	0.696	
		Weekend warrior	29	3 (10.3)	7.0	1.08 (0.18~6.36)	0.933	
		Trend test	93	10 (10.8)	7.2	1.04 (0.42~2.57)	0.94	
	≤1-2 times/week							
		Inactive	273	31 (11.4)	7.5	1(Ref)		
		Regularly active	106	4 (3.8)	2.5	0.39 (0.13~1.13)	0.083	
		Weekend warrior	273	14 (5.1)	3.3	0.49 (0.25~0.94)	0.032	
		Trend test	652	49 (7.5)	4.9	0.68 (0.49~0.96)	0.028	
	≥3 times/week							
		Inactive	220	24 (10.9)	7.2	1(Ref)		
		Regularly active	120	5 (4.2)	2.7	0.5 (0.18~1.35)	0.17	
		Weekend warrior	263	13 (4.9)	3.2	0.47 (0.23~0.94)	0.033	
		Trend test	603	42 (7)	4.6	0.68 (0.47~0.97)	0.032	
Education								0.42
	College or university degree							
		Inactive	177	19 (10.7)	7.1	1(Ref)		

		Total	N event%	Incidence rate	HR (95%CI)	P value	P for interaction
	Regularly active	115	5 (4.3)	2.8	0.46 (0.17~1.27)	0.132	
	Weekend warrior	272	10 (3.7)	1.2	0.35 (0.16~0.78)	0.011	
	Trend test	564	34 (6)	3.9	0.59 (0.39~0.88)	0.01	
Others							
	Inactive	365	42 (11.5)	7.6	1(Ref)		
	Regularly active	126	5 (4)	2.6	0.42 (0.16~1.08)	0.073	
	Weekend warrior	293	20 (6.8)	4.5	0.63 (0.36~1.11)	0.109	
	Trend test	784	67 (8.5)	5.6	0.78 (0.59~1.04)	0.093	
BMI							0.546
< 24							
	Inactive	74	5 (6.8)	4.4	1(Ref)		
	Regularly active	55	3 (5.5)	3.6	0.63 (0.14~2.84)	0.552	
	Weekend warrior	123	5 (4.1)	2.7	0.31 (0.08~1.24)	0.098	
	Trend test	252	13 (5.2)	3.4	0.55 (0.28~1.11)	0.095	
> 24							
	Inactive	468	56 (12)	7.9	1(Ref)		
	Regularly active	186	7 (3.8)	2.5	0.39 (0.18~0.88)	0.023	
	Weekend warrior	442	25 (5.7)	3.7	0.53 (0.33~0.87)	0.012	
	Trend test	1096	88 (8)	5.3	0.71 (0.55~0.92)	0.009	

HR, hazard ratio; *CI*: confidence interval; *BMI*, Body mass index;

Supplementary Table 6: Association between physical activity pattern and all-cause mortality in individuals with kidney stone (additionally adjusting for Water intake, Blood Creatinine, Potassium in urine, Sodium in urine, Cholesterol, Triglycerides, Total protein)

Variable	Total	N event%	Incidence rate	Model1		Model2	
			(per 1000 person-year)	HR (95%CI)	P value	HR (95%CI)	P value
Inactive	542	61 (11.3)	7.4	1(Ref)		1(Ref)	

Regularly active	241	10 (4.1)	2.7	0.36 (0.19~0.71)	0.003	0.54 (0.26~1.14)	0.108
Weekend warrior	565	30 (5.3)	3.5	0.46 (0.3~0.71)	0.001	0.44 (0.25~0.75)	0.003
Trend test	1348	101 (7.5)	4.9	0.66 (0.52~0.82)	<0.001	0.66 (0.5~0.86)	0.003

HR, hazard ratio; *CI*: confidence interval.

Weekend warrior pattern was defined as MVPA ≥ 150 min/week (guideline-recommended) with ≥ 50% concentrated on 1–2 days per week; regularly active pattern was defined as MVPA ≥ 150 min/week with ≥ 50% distributed on > 2 days per week; inactive group was defined as MVPA < 150 min/week

Model 1: unadjusted.

Model 2: adjusted for age, sex, race and ethnicity+ BMI, Townsend deprivation index, Smoking status, Frequency of alcohol drinking, education+ additionally adjusting for Water intake, Blood Creatinine, Potassium in urine, Sodium in urine, Cholesterol, Triglycerides, Total protein**)**

Supplementary Table 7: Association between physical activity pattern and all-cause mortality in individual with kidney stone (after imputing missing values for all covariates using multiple imputation method)

**Outcome**	**Physical activity pattern**	**Total**	**N event%**	**Incidence rate (per 1000 person-year)**	**HR(95%CI)**	***P value***
All-cause mortality	Inactive	561	64 (11.4)	7.5	1(Ref)	
	Regularly active	245	10 (4.1)	2.7	0.44 (0.22~0.86)	0.017
	Weekend warrior	582	30 (5.2)	3.4	0.49 (0.31~0.77)	0.002
	Trend test	1388	104 (7.5)	4.9	0.69 (0.54~0.87)	0.002

HR, hazard ratio; *CI*: confidence interval

Note: Weekend warrior pattern was defined as MVPA ≥ 150 min/week (guideline-recommended) with ≥ 50% concentrated on 1–2 days per week; regularly active pattern was defined as MVPA ≥ 150 min/week with ≥ 50% distributed on > 2 days per week; inactive group was defined as MVPA < 150 min/week

*Adjusted for age, sex, race and ethnicity, BMI, Townsend deprivation index, Smoking status, Frequency of alcohol drinking, education

Supplementary Table 8: Association between physical activity patterns and all-cause mortality stratified by baseline kidney stone status

Outcome	Physical activity pattern	HR (95% CI)			*P for interaction*
		Whole cohort	Individuals with kidney stone	Individuals without kidney stone	
All-cause mortality	Inactive	1(Ref)	1(Ref)	1(Ref)	0.314
	Regularly active	0.73 (0.67~0.79)	0.46 (0.23~0.91)	0.73 (0.68~0.79)	
	Weekend warrior	0.69 (0.65~0.73)	0.51 (0.32~0.82)	0.69 (0.65~0.74)	
	Trend test	0.83 (0.8~0.86)	0.71 (0.56~0.9)	0.83 (0.81~0.86)	

HR, hazard ratio; *CI*: confidence interval

Supplementary Table 9. Sensitivity analysis of the associations between physical activity patterns and all-cause mortality among individuals with kidney stones, with follow-up starting at accelerometer assessment

Outcome	Total	N event%	Model1		Model2		Model3	
			HR (95%CI)	*P value*	HR (95%CI)	*P value*	HR (95%CI)	*P value*
Inactive	542	61 (11.3)	1.00 (Reference)	…	1.00 (Reference)	…	1.00 (Reference)	…
Regularly active	241	10 (4.1)	0.35 (0.18~0.69)	0.002	0.36 (0.18~0.71)	0.003	0.44 (0.22~0.88)	0.019
Weekend warrior	565	30 (5.3)	0.46 (0.29~0.71)	< 0.001	0.43 (0.27~0.67)	<0.001	0.51 (0.32~0.81)	0.004
Trend test	1348	101 (7.5)	0.65 (0.52~0.82)	<0.001	0.64 (0.5~0.8)	<0.001	0.7 (0.55~0.89)	0.004

Weekend warrior pattern was defined as MVPA ≥ 150 min/week (guideline-recommended) with ≥ 50% concentrated on 1–2 days per week; regularly active pattern was defined as MVPA ≥ 150 min/week with ≥ 50% distributed on > 2 days per week; inactive group was defined as MVPA < 150 min/week

Model 1: unadjusted.

Model 2: adjusted for age, sex, race and ethnicity.

Model 3: adjusted for model 2+ BMI, Townsend deprivation index, Smoking status, Frequency of alcohol drinking, education

Supplementary Table 10. Sensitivity analysis of the associations between physical activity patterns and all-cause mortality after excluding deaths within 1 year of accelerometer assessment

Outcome	Total	N event%	Model1		Model2		Model3	
			HR (95%CI)	*P value*	HR (95%CI)	*P value*	HR (95%CI)	*P value*
Inactive	541	60 (11.1)	1.00 (Reference)	…	1.00 (Reference)	…	1.00 (Reference)	…
Regularly active	241	10 (4.1)	0.36 (0.18~0.7)	0.003	0.37 (0.19~0.72)	0.003	0.45(0.23~0.90)	0.023
Weekend warrior	565	30 (5.3)	0.46 (0.30~0.72	0.001	0.44 (0.28~0.68)	<0.001	0.52 (0.32~0.82)	0.005
Trend test	1347	100 (7.4)	0.66(0.52~0.83)	<0.001	0.64 (0.51~0.81)	<0.001	0.71 (0.56~0.90)	0.005

Model 1: unadjusted.

Model 2: adjusted for age, sex, race and ethnicity.

Model 3: adjusted for model 2+ BMI, Townsend deprivation index, Smoking status, Frequency of alcohol drinking, education

Supplementary Table 11. Sensitivity analysis of the associations between physical activity patterns and all-cause mortality after excluding deaths within 2 years of accelerometer assessment

Outcome	Total	N event%	Model1		Model2		Model3	
			HR (95％CI)	*P value*	HR (95％CI)	*P value*	HR (95％CI)	*P value*
Inactive	537	56 (10.4)	1.00 (Reference)	…	1.00 (Reference)	…	1.00 (Reference)	…
Regularly active	241	10 (4.1)	0.38 (0.20~0.75)	0.005	0.39 (0.20~0.77)	0.007	0.48(0.24~0.96)	0.037
Weekend warrior	564	29 (5.1)	0.48 (0.31~0.75)	0.001	0.46 (0.29~0.72)	0.001	0.54 (0.34~0.87)	0.011
Trend test	1342	95 (7.1)	0.67(0.53~0.85)	0.001	0.66 (0.52~0.84)	0.001	0.73 (0.57~0.93)	0.01

Model 1: unadjusted.

Model 2: adjusted for age, sex, race and ethnicity.

Model 3: adjusted for model 2+ BMI, Townsend deprivation index, Smoking status, Frequency of alcohol drinking, education
